# Immunometabolism and mitochondria in inflammatory bowel disease: a role for therapeutic intervention?

**DOI:** 10.1242/dmm.050895

**Published:** 2024-10-17

**Authors:** Claire E. Adams, Duncan G. Rutherford, Gareth R. Jones, Gwo-tzer Ho

**Affiliations:** Gut Research Unit, Centre for Inflammation Research, Institute for Regeneration and Repair, University of Edinburgh, Edinburgh EH16 4UU, UK

## Abstract

Inflammatory bowel diseases (IBDs), incurable conditions characterised by recurrent episodes of immune-mediated gut inflammation and damage of unknown aetiology, are common. Current advanced therapies target key leukocyte-trafficking and cytokine-signalling hubs but are only effective in 50% of patients. With growing evidence of mitochondrial dysfunction in IBD and advances in our understanding of the role of metabolism in inflammation, we provide an overview of novel metabolic approaches to IBD therapy, challenging the current ‘therapeutic ceiling’, identifying critical pathways for intervention and re-imagining metabolic biomarkers for the 21st century.

## Teaser

**Summary:** We provide an overview of mitochondrial and metabolic aberrations encountered in inflammatory bowel disease (IBD) and discuss potential immunometabolic therapies as emerging novel approaches for IBD.

## Introduction

Inflammatory bowel diseases (IBDs), including ulcerative colitis (UC) and Crohn's disease (CD), are incurable conditions that affect 8-10 million people worldwide, with a growing global prevalence (Wang et al., 2023). Fundamentally, both conditions are characterised by immune-mediated chronic inflammation affecting the gut, with strong genetic susceptibility and environmental influences, such as diet and lifestyle factors. UC and CD are associated with a lifelong risk of disease relapse, and often unremitting chronic gut inflammation and tissue damage without medical intervention. Glucocorticoids and general immunosuppressants, such as thiopurines and methotrexate, form the traditional mainstay of medical treatments for IBD. Recently, there have been significant advances in developing more specific approaches that target pro-inflammatory cytokines and integrins using monoclonal antibodies (anti-TNF, anti-α_4_β_7_, anti-IL23p40 and anti-IL23p19) and small molecules in immune signalling pathways, such as Janus kinase (JAK) inhibitors (see Glossary, Box 1) and sphingosine-1 phosphate (S1P) modulators (Box 1). Beyond IBD, these widely available drugs have transformed the clinical landscape of many immune-mediated diseases, such as rheumatoid arthritis and psoriasis. It is noteworthy that they are targeted at the downstream inflammatory response in IBD, and the causative mechanisms remain poorly understood. Furthermore, in severe IBD, they are effective in only 50% of patients, and their ability to completely heal the intestinal mucosa (Box 1) remains poor.

**Figure:**
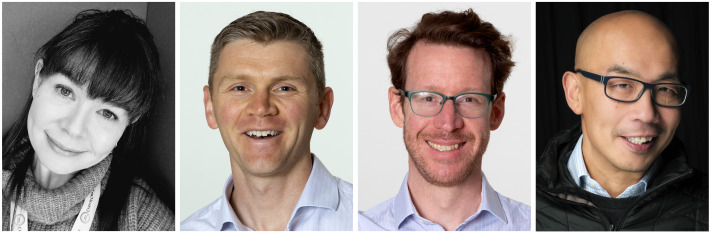
Claire E. Adams, Duncan G. Rutherford, Gareth R. Jones and Gwo-tzer Ho (left to right)

**Box 1. Glossary**

**Antimicrobial peptides:** evolutionarily conserved short-chain peptides with anti-microbial and immunomodulatory properties.

**Damage-associated molecular patterns (DAMPs):** small molecules released from stressed/damaged/dying cells that act as danger signals to the innate immune system, activating it in cases of sterile inflammation.

**Electron transport chain (ETC):** a series of protein complexes (I-IV) embedded within the inner mitochondrial membrane. Here, electrons are transported, creating a transmembrane electrochemical proton (H^+^) gradient. ATP synthase (complex 4) is the site of oxidative phosphorylation at which this channel allows H^+^ ions back across the gradient, generating ATP.

**Glycolysis:** the ten-step enzymatic metabolism of glucose that occurs in the cytosol of all cells and can be aerobic (leading to formation of pyruvate) or anaerobic (leading to formation of lactate). Although a quick process, it results in only a net of two ATP molecules per molecule of glucose, and so it is a relatively inefficient way for a cell to generate energy.

**Hypoxia-inducible factor 1-α (HIF1A):** a key transcription factor that regulates a cell's response to changes in oxygen tension and is also a master regulator of cellular metabolism.

**Immunometabolism:** an emerging field of immunology research that can be described as the study of immune cell metabolic processes and metabolic intermediaries in health and in stress.

**Intestinal mucosa:** comprising the lumen-facing epithelial cell layer, underlying lamina propria, and outer muscularis mucosa. The intact barrier allows water and nutrient uptake, promotes immune tolerance to commensals and food antigens, and prevents pathogens and toxins from accessing the systemic circulation.

**Janus kinase (JAK) inhibitors:** a class of drug used to treat many chronic autoimmune conditions. They act by preventing JAK (a cytoplasmic non-receptor tyrosine kinase) phosphorylating, and thus activating, STAT proteins, disrupting downstream cytokine signalling.

**Metabolic re-programming:** when cells alter their fundamental metabolic pathways to adapt to their environment. This is a hallmark feature of cancer cells to promote a pro-survival tumour microenvironment and is increasingly recognised in chronic inflammation.

**NOD-, LRR- and pyrin domain-containing protein 3 (NLRP3):** a three-protein structure found in the cytosol of immune cells. It can be activated by cell stress from a wide variety of sources – from bacterial, viral or fungal infections to sterile inflammation and environmental toxins. NLRP3 activation induces inflammasome formation, which leads to caspase activation and, in turn, secretion of active forms of IL1β, IL18 and gasdermin D.

**Nuclear factor erythroid 2-related factor 2 (NRF2):** a transcription factor that regulates antioxidant enzymes, e.g. superoxide dismutase, glutathione peroxidase.

**Oxidative phosphorylation:** collective term for the redox reactions occurring in the ETC and chemiosmosis. It occurs in the mitochondria, requires oxygen and generates the majority of cellular ATP, between 30 and 32 ATP molecules per molecule of glucose.

**Pentose phosphate pathway:** a ‘shunt’ from glycolysis; this cytosolic reaction generates pentose phosphates needed for DNA and RNA synthesis, but no ATP.

**Sphingosine-1 phosphate (S1P) modulators:** an emerging class of drugs used in some autoimmune conditions. They prevent lymphocytes from sensing S1P, limiting their migration from lymph nodes to inflamed tissues.

**Stimulator of interferon gene (STING):** a transmembrane protein found on the endoplasmic reticulum in cells and a key mediator of the type 1 interferon response to DNA released by dying cells, bacteria or viruses.

**Toll-like receptor (TLR):** transmembrane protein, of which ten subtypes have been identified in humans at the time of writing. They can be present on the cell plasma membrane of antigen-processing cells or can be intracellular. Molecules from pathogens bind to TLRs – e.g. lipopolysaccharide to TLR4 and mitochondrial DNA to TLR9 – inducing an innate immune response by initiation of a downstream signalling cascade.

**Tricarboxylic acid (TCA) cycle:** otherwise known as the citric acid cycle or Krebs’ cycle, a series of biochemical reactions to release the energy stored in nutrients through the oxidation of acetyl-CoA derived from carbohydrates, fats and proteins. It generates electrons, which are transferred by NAD and FADH donated to the ETC.

**Xenobiotics:** chemicals or toxins that are foreign to the body. They are usually metabolised hepatically, but can mount an immune response.

There have been major advances in understanding how metabolism, in particular the mitochondria, governs the immune response in homeostasis and disease states (Murphy and O'Neill, 2024) and how it drives the action of glucocorticoids, the longstanding anti-inflammatory treatment in human diseases (Auger et al., 2024). This commentary focuses on the role of immunometabolism (Box 1) as a tractable and hitherto novel therapeutic avenue in IBD.

## The relevance of immunometabolism in IBD

The gastrointestinal tract is a highly metabolically active organ with an epithelial lining that is replenished every 4-6 days from intestinal stem cells (iSCs). It must balance energy-demanding physiological functions, such as nutrient absorption and gut repair, while maintaining a robust immune defence that involves the production of antimicrobial peptides (Box 1), mucus and immunoglobulins. Here also lies one of the most immune-rich tissue environments, with macrophages, dendritic cells, B cells, T cells, natural killer cells, innate lymphoid and plasma cells that provide a sophisticated and highly complex defence at the interface with trillions of gut bacteria. They function as a finely tuned biological system that senses the changes within the gut luminal environment. Tissue-resident cells, such as macrophages, are omnipresent, and there are also those that respond, differentiate or traffic to the gut in response to danger, such as monocytes, neutrophils and effector T cells, and others that maintain homeostasis, such as regulatory T cells. Thus, immune cell metabolism that is required to ‘fuel’ these dynamic needs and inflammatory potential in the gut are intricately linked. Pertinently, distinct metabolic changes in the gut can profoundly influence the tissue's immune response in a primary manner (Buck et al., 2017).

The regulation of metabolism is dependent on intertwined biological networks including nutrient and energy sensor pathways, such as mechanistic target of rapamycin (mTOR) and AMP-activated protein kinase (AMPK) (Saxton and Sabatini, 2017). In broad terms, this metabolic system can be actively switched to anti- or pro-inflammatory functions depending on the biological need. *In vitro* work has demonstrated that both innate and adaptive immune cells upregulate glycolysis (Box 1) in the presence of pro-inflammatory stimuli to accommodate rapid generation of ATP, and downregulate oxidative phosphorylation (Box 1), which is associated with an anti-inflammatory immune cell phenotype and function. Accompanying metabolic changes in immune cells during inflammation include an increase in substrate flux through the pentose phosphate pathway (Box 1) and ‘breaks’ in the tricarboxylic acid (TCA) cycle (Box 1), leading to the accumulation of metabolites that are known to have immunoregulatory functions – succinate and citrate. These are termed ‘immuno-metabolites’ and have been shown to act as co-factors for metabolic enzyme reactions, mediate post-translational modifications, signal between cells as cytokines would (metabokines), and even drive immune cell phenotype and function (Murphy and O'Neill, 2018). The gut is also a source of molecules that arise from the metabolism of host microbiota, such as short-chain fatty acids, that are essential metabolic fuel sources and have major local and systemic immunomodulatory properties (Mann et al., 2024).

The mitochondrion provides the intracellular platform for many key metabolic functions (Monzel et al., 2023). Of interest, mitochondria can remodel their shape by fission and fusion to become active controllers of cellular metabolism during health and disease (Nunnari and Suomalainen, 2012). Within the gut, mitochondria are uniquely exposed to high levels of potentially harmful bacteria, immune-active ligands, toxins and xenobiotics (Box 1). Hence, there is more mitochondrial stress and potential for damage in the gut than in other tissues and organs.

**Fig. 1.**
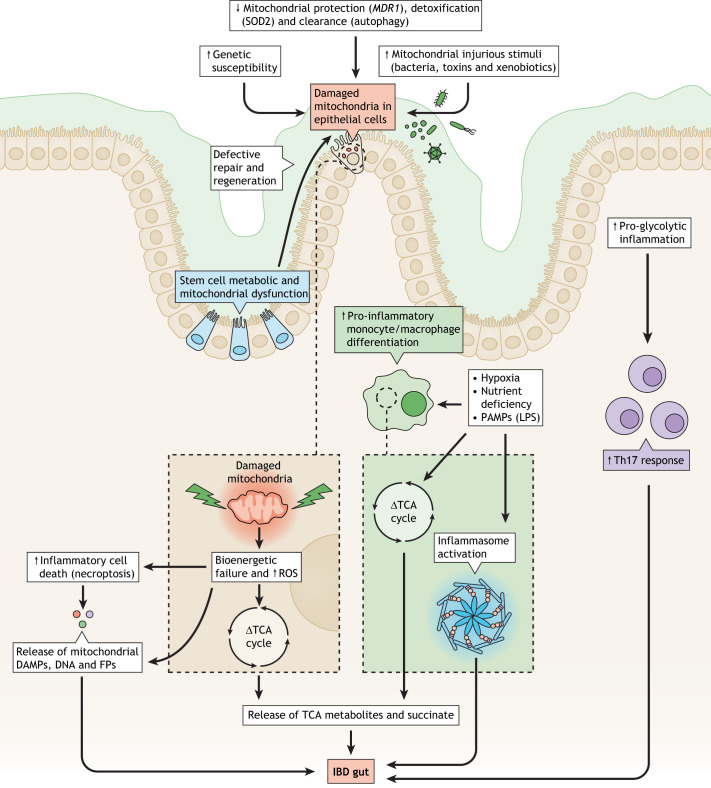
**Mechanisms and immunometabolic therapeutic targets in the gut and IBD.** Pathological factors, such as genetic susceptibility, decreased homeostatic pathways, increased mitochondrial injurious stimuli, hypoxia, nutritional factors and PAMPs, can all lead to mitochondrial and metabolic dysfunction and inflammatory processes in relevant intestinal cells (epithelial, intestinal stem and immune cells). Metabolic and mitochondrial dysfunction in intestinal stem cells causes defective repair and regeneration of the epithelial layer and contributes to metabolic and mitochondrial dysfunction in intestinal epithelial cells. The resultant pro-inflammatory processes include increased ROS, inflammatory cell death, inflammasome activation, and release of inflammatory metabolites and DAMPs. DAMP, damage associated molecular pattern; FP, mitochondrial formylated peptide; IBD, inflammatory bowel disease; LPS, lipopolysaccharide; *MDR1*, multidrug resistance 1 (also known as *ABCB1*); PAMP, pathogen associated molecular pattern; ROS, reactive oxygen species; SOD2, superoxide dismutase 2; TCA, tricarboxylic acid cycle; Th, T helper.

In addition to mitochondrial dysfunction, the inflamed IBD gut imposes distinct immunological, nutritional, hypoxic and metabolic demands on the immune, mesenchymal and epithelial cell populations that can lead to metabolic re-programming (Box 1) (see Fig. 1) (Friedrich et al., 2019; Colgan et al., 2010). The majority of immunometabolism studies have so far been predominantly based on mouse systems and little on disease states such as IBD. However, there are early observational studies in humans to show that de-regulation of metabolism is evident in IBD (Macias-Ceja et al., 2019; Kolho et al., 2017).

Genome-wide association studies indicate that ∼5% of IBD susceptibility genes have important roles in regulating mitochondrial function

## Mitochondrial dysfunction in IBD and its consequences

There is growing evidence of mitochondrial dysfunction in IBD. Genome-wide association studies indicate that ∼5% of IBD susceptibility genes have important roles in regulating mitochondrial function (Ho and Theiss, 2022). This is strongly supported by transcriptomic data pointing towards a downregulation of mitochondrial- and nuclear-encoded mitochondrial genes in the IBD gut (Haberman et al., 2019), and mitochondrial gene polymorphisms associated with energy deficiency (Dankowski et al., 2016). Mitochondria can also be directly damaged in the IBD gut (Mottawea et al., 2016), which is further amplified by loss of the protective mechanisms for the mitochondria in IBD (Ho et al., 2018). Consequentially, this can result in the accumulation of damaged mitochondria and the generation of pro-inflammatory mitochondrial reactive oxygen species (mROS). Although excessive reactive oxygen species (ROS) generation has a pro-inflammatory effect, a baseline level of ROS is generated from oxidative phosphorylation and is required for epithelial differentiation (Rodriguez-Coleman et al., 2017) and activation of antigen-specific T cells (Sena et al., 2013). Moreover, ROS deficiency can lead to epithelial barrier disruption and increase susceptibility to colitis (Aviello et al., 2019; Hsu et al., 2023). Nevertheless, failed clearance or repair of damaged mitochondria and excessive mROS can lead to their accumulation and the release of mitochondrial constituents (DNA, formylated peptides), which serve as damage-associated molecular patterns (DAMPs; Box 1) that can further perpetuate gut inflammation (Boyapati et al., 2018).

Bioenergetic failure due to mitochondrial dysfunction renders the epithelial lining more susceptible to damage and poor healing (Bar et al., 2013). Furthermore, mitochondrial damage in iSCs can be propagated and imprinted in the gut epithelial phenotype, thus influencing how the epithelium metabolises, regenerates and repairs (Smith et al., 2020). Finally, mitochondrial damage and metabolic reprogramming of adaptive and innate immune cells are intimately linked (see next section for more detail). These lines of evidence point to mitochondrial dysfunction as a pro-inflammatory factor that can perpetuate chronic inflammation and become a major barrier for mucosal healing even when immunosuppressive drugs are used.

The current ‘therapeutic ceiling’ of 50% of patients responding to advanced immune therapies suggests that there remains a major unmet need and that the direct targeting of the inflammatory pathways alone is insufficient

## Immunometabolism opens new therapeutic avenues for IBD

The current ‘therapeutic ceiling’ of 50% of patients responding to advanced immune therapies suggests that there remains a major unmet need and that the direct targeting of the inflammatory pathways alone is insufficient (Ho et al., 2022). Here, the nascent field of immunometabolism will be highly relevant, with many potential ‘druggable’ pathways and perhaps the option of repurposing existing therapeutics. These therapeutic approaches are based on the concept of beneficial modulation of the complex inflammatory IBD gut that promotes pro-resolution and repair functions of relevant immune cells (macrophage and T cells), in addition to improving the fitness of the epithelial cells during mucosal healing. Therefore, there are several translational scientific opportunities, as described below.

### Modulating mitochondrial function and metabolism

Many pre-clinical studies relevant to autoimmune diseases show that inhibiting mitochondrial ROS can be beneficial in inflammatory states, as exogenously inducing mROS worsened acute colitis in a mouse model, whereas inhibition of mROS attenuated disease (Lood et al., 2016; Jackson et al., 2020; Ho et al., 2018). The necessary translational step is now to explore whether such a simple approach can be efficacious in human inflammatory disease. In this context, the MARVEL study (www.marvelstudy.uk; NCT04276740) in the UK is currently investigating the use of mitochondrial antioxidant MitoQ as an adjunct to steroid therapy for UC (Gwyer-Findlay et al., 2021).

Nod-like receptor-1 (NLRX1) is a negative regulator of inflammation (Leber et al., 2018) and is found on the outer mitochondrial membrane of most cells, including epithelial and immune cells. Its deficiency in mouse models of colitis correlates with more severe disease and a pro-inflammatory, pro-glycolytic T-cell phenotype (Leber et al., 2017). NX-13, an oral agonist of NLRX1, showed promising results in ameliorating disease in pre-clinical models (Leber et al., 2019) and is currently being studied in phase 2 trials for UC (NEXUS trial, NCT05785715).

Targeting mitochondrial dynamics is a further avenue, whereby inhibiting mitochondrial fission through the administration of P110, a compound that prevents the binding of two key mitochondrial fission proteins, has been shown to improve chemically induced mouse colitis (Mancini et al., 2020).

### Targeting immunometabolites

The immunometabolite succinate is of particular interest, as levels of both it and its receptor SUCNR1 are increased in IBD (Fremder et al., 2021; Macias-Ceja et al., 2019). Furthermore, pro-inflammatory macrophages have high levels of succinate (Diskin et al., 2021). *In vitro*, succinate has been shown to be released from macrophages and then signal via SUCNR1 present on dendritic cells and macrophages, acting as a pro-inflammatory mediator (de Goede et al., 2019). Succinate also inhibits the enzymes responsible for degrading hypoxia-inducible factor 1-α (HIF1A; Box 1), leading to its stabilisation even in normoxia. This can lead to increased IL1β production and reversal of the role of the electron transport chain (ETC; Box 1) from ATP generation to ROS production (Tannahill et al., 2013). Countering the effects of HIF1A and succinate, aconitate decarboxylase 1 (ACOD1) acts as a regulator of the inflammatory response to infection and other inflammatory stimuli. ACOD1 is the obligate source of the metabolite itaconate, which stabilises nuclear factor erythroid 2-related factor 2 (NRF2; also known as NFE2L2; Box 1). NRF2 attenuates NOD-, LRR- and pyrin-domain containing protein 3 (NLRP3; Box 1)-driven inflammation and stimulator of interferon gene (STING; also known as STING1; Box 1)-modulated IFN responses, promoting anti-inflammatory and anti-oxidant effects (Michelucci et al., 2013; Naujoks et al., 2016; Lampropoulou et al., 2016). During healthy conditions, itaconate is present in negligible quantities. Its expression can be induced by a suite of factors, including Toll-like receptor (TLR; Box 1)4 ligands, type I and II IFN, bacteria and viruses, although much of this evidence is derived from *in vitro* studies of stimulated murine blood leukocytes (Lampropoulou et al., 2016; Michelucci et al., 2013; Naujoks et al., 2016). Thus, emerging evidence places itaconate as a major anti-inflammatory metabolite, with 4-octyl itaconate (4-OI), an analogue of itaconate, being explored currently in phase 1 trials for IBD (https://www.sitryx.com/pipeline#pipeline).

### Improving metabolic fitness in IBD gut repair

With data implicating mitochondrial dysfunction in iSCs (Parikh et al., 2019; Smillie et al., 2019), future pro-repair approaches have been explored in intestinal organoids, including strategies to repair metabolic defects at the mitochondrial level, particularly for the purpose of treating severe IBD (Rutherford and Ho, 2023). Of interest, the ketone body β-hydroxybutyrate has been shown to promote an anti-inflammatory macrophage phenotype and proliferation of intestinal epithelial cells, which improved experimental colitis in a mouse model (Cheng et al., 2019; Huang et al., 2022).

## Clinical approaches to immunometabolism in IBD – ‘low-hanging fruits’

There are now many IBD randomised clinical trials (phases II-IV), but it will be very difficult to envisage future novel approaches being more successful than advanced immune therapies, such as anti-TNF and JAK inhibitors, in the clinical trial setting. Our view is that immunometabolic therapies will be used as an adjunct rather than primary treatment, with future clinical trials designed in this context. ‘Multidrug resistant’ IBD patients that rapidly cycle through different immune therapies or those that cannot achieve satisfactory gut mucosal healing are good initial clinical cohorts to investigate and define immunometabolic profiles. In CD, there is a wealth of evidence implicating macrophage dysfunction in disease aetiology, including defective bacterial killing, monocyte–macrophage differentiation and mucosal healing. Here, macrophage-targeted drugs may be the ‘low-hanging fruit’ for immunometabolic therapies (Hegarty et al., 2023) (Box 2). The paradigm of anti- versus pro-inflammatory metabolism similarly applies to T-cell function, with a wealth of data demonstrating its importance in infection and cancer (reviewed in detail in Reina-Campos et al., 2021), and, more specifically, in CD4^+^ T cells (Baixauli et al., 2022) and T-helper (Th)17 responses (Johnson et al., 2018) that are highly relevant to IBD. It is beyond the scope of our commentary to provide an in-depth review of this; however, clinically available IL23–Th17-targeted therapies for IBD, such as ustekinumab and risankizumab, provide an opportunity to carry out human experimental studies and provide more mechanistic insights into the impact of these therapies on gut T cells. For example, future studies could interrogate how immunometabolism changes in gut T cells before and after therapy in patients.

**Box 2. Macrophages in inflammatory bowel disease (IBD) – a case example for immunometabolic intervention**

Macrophages are the most abundant immune cell in the intestinal mucosa and are largely continuously replenished by circulating classical monocytes. In health, these monocytes differentiate into macrophages with an anti-inflammatory, pro-homeostatic phenotype, and play key roles in immune tolerance to food antigens and commensal organisms. In IBD, loss of barrier function and subsequent translocation of pro-inflammatory stimuli result in the recruitment of monocytes that differentiate into aggressively pro-inflammatory macrophages that persist in the IBD gut and shape the pathogenic T-effector responses. These macrophages and many of their products, such as TNF, IL1β and oncostatin M, activate and perpetuate gut inflammation in IBD, including the recruitment of other immune cells such as neutrophils. Current treatments such as anti-TNF, anti-α_4_β_7_ and anti-IL23 therapies target pro-inflammatory pathways in innate and adaptive immune cells (Hegarty et al., 2023).

It is clear that altered metabolism in macrophages plays a role in IBD pathogenesis, but there are key questions outstanding for effective immunometabolic interventions that target these cells:

(1) Is the pro-inflammatory macrophage function amenable to specific pharmacologic modulation?
(2) Will this increase the likelihood of complete mucosal healing in IBD?
(3) Can we stratify immunometabolic therapy based on functional phenotyping of IBD macrophages?

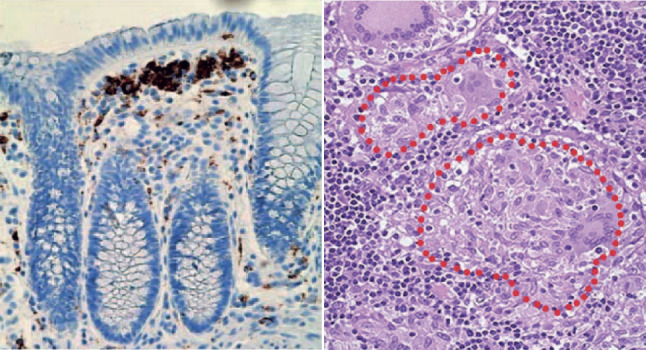

Left: macrophages in the human gut shown in brown (CD68–DAB). Right: the presence of granuloma in the human IBD gut; red dotted lines outline aggregates of macrophages in Crohn's disease.

## Metabolic biomarkers to stratify future therapy in IBD

Given the heterogeneity of IBD and mucosal inflammatory milieu – with so many factors that can affect mitochondria function and metabolism – finding metabolic biomarkers that can stratify patients to the relevant immunometabolic therapies is crucial. Experimental approaches to study immunometabolism are now increasingly advanced, from using functional metabolomic and flow-cytometry panels, such as single-cell energetic metabolism by profiling translation inhibition (SCENITH) (Arguello et al., 2020) and metabolism by flow cytometry (MetFlow) (Ahl et al., 2020), to single-cell analyses (Voss et al., 2021). However, they need more realistic consideration of how to apply them in larger-scale populations and how closely patient samples used for the analyses recapitulate *in vivo* situations, especially in the unique hypoxic and metabolic environment of the gut. High levels of mitochondrial DNA in the blood – often seen in the context of extensive tissue damage, such as trauma or sepsis, and in immune-mediated conditions, such as systemic lupus erythematosus – have also been observed in active IBD (Lood et al., 2016; Zhang et al., 2010; Boyapati et al., 2018). Measuring circulating mitochondrial DNA (and other DAMPs) is currently being investigated as a surrogate for mitochondria dysfunction (www.musicstudy.uk; NCT04760964). Simple high-throughput screening of TCA metabolites is now also needed, especially with the advent of itaconate mimetics. The addition of metabolic read-outs is increasingly considered in large multi-omic studies in IBD.

Immunometabolism is a rapidly advancing field. In IBD, this is a translational area of significant interest with the potential to break the ‘therapeutic ceiling’ in treating IBD gut inflammation.

## Conclusion

Immunometabolism is a rapidly advancing field. In IBD, this is a translational area of significant interest with the potential to break the ‘therapeutic ceiling’ in treating IBD gut inflammation. Beyond this, there is also significant potential for other vital patient-centred outcomes, such as fatigue, and more widely for other immune-mediated conditions, such as rheumatoid arthritis, where such a novel approach will be highly relevant (Schett et al., 2021).

## References

[DMM050895C1] Ahl, P. J., Hopkins, R. A., Xiang, W. W., Au, B., Kaliaperumal, N., Fairhurst, A. M. and Connolly, J. E. (2020). Met-Flow, a strategy for single-cell metabolic analysis highlights dynamic changes in immune subpopulations. *Commun. Biol.* 3, 305. 10.1038/s42003-020-1027-9 32533056 PMC7292829

[DMM050895C2] Arguello, R. J., Combes, A. J., Char, R., Gigan, J. P., Baaziz, A. I., Bousiquot, E., Camosseto, V., Samad, B., Tsui, J., Yan, P. et al. (2020). SCENITH: a flow cytometry-based method to functionally profile energy metabolism with single-cell resolution. *Cell Metab.* 32, 1063-1075.e7. 10.1016/j.cmet.2020.11.007 33264598 PMC8407169

[DMM050895C3] Auger, J. P., Zimmermann, M., Faas, M., Stifel, U., Chambers, D., Krishnacoumar, B., Taudte, R. V., Grund, C., Erdmann, G., Scholtysek, C. et al. (2024). Metabolic rewiring promotes anti-inflammatory effects of glucocorticoids. *Nature* 629, 184-192. 10.1038/s41586-024-07282-7 38600378

[DMM050895C48] Aviello, G., Singh, A. K., O'Neill, S., Conroy, E., Gallagher, W., D'Agostino, G., Walker, A. W., Bourke, B., Scholz, D. and Knaus, U. G. (2019). Colitis susceptibility in mice with reactive oxygen species deficiency is mediated by mucus barrier and immune defense defects. *Mucosal Immunol.* 12, 1316-1326. 10.1038/s41385-019-0205-x 31554901

[DMM050895C4] Baixauli, F., Piletic, K., Puleston, D. J., Villa, M., Field, C. S., Flachsmann, L. J., Quintana, A., Rana, N., Edwards-Hicks, J., Matsushita, M. et al. (2022). An LKB1-mitochondria axis controls T(H)17 effector function. *Nature* 610, 555-561. 10.1038/s41586-022-05264-1 36171294 PMC9844518

[DMM050895C5] Bar, F., Bochmann, W., Widok, A., Von Medem, K., Pagel, R., Hirose, M., Yu, X., Kalies, K., Konig, P., Bohm, R. et al. (2013). Mitochondrial gene polymorphisms that protect mice from colitis. *Gastroenterology* 145, 1055-1063. e3. 10.1053/j.gastro.2013.07.015 23872498

[DMM050895C6] Boyapati, R. K., Dorward, D. A., Tamborska, A., Kalla, R., Ventham, N. T., Doherty, M. K., Whitfield, P. D., Gray, M., Loane, J., Rossi, A. G. et al. (2018). Mitochondrial DNA is a pro-inflammatory damage-associated molecular pattern released during active IBD. *Inflamm. Bowel Dis.* 24, 2113-2122. 10.1093/ibd/izy095 29718255 PMC7301773

[DMM050895C7] Buck, M. D., Sowell, R. T., Kaech, S. M. and Pearce, E. L. (2017). Metabolic instruction of immunity. *Cell* 169, 570-586. 10.1016/j.cell.2017.04.004 28475890 PMC5648021

[DMM050895C8] Cheng, C. W., Biton, M., Haber, A. L., Gunduz, N., Eng, G., Gaynor, L. T., Tripathi, S., Calibasi-Kocal, G., Rickelt, S., Butty, V. L. et al. (2019). Ketone body signaling mediates intestinal stem cell homeostasis and adaptation to diet. *Cell* 178, 1115-1131.e15. 10.1016/j.cell.2019.07.048 31442404 PMC6732196

[DMM050895C51] Colgan, S. P. and Taylor, C. T. (2010). Hypoxia: an alarm signal during intestinal inflammation. *Nat. Rev. Gastroenterol. Hepatol.* 7, 281-287. 10.1038/nrgastro.2010.39 20368740 PMC4077542

[DMM050895C9] Dankowski, T., Schroder, T., Moller, S., Yu, X., Ellinghaus, D., Bar, F., Fellermann, K., Lehnert, H., Schreiber, S., Franke, A. et al. (2016). Male-specific association between MT-ND4 11719 A/G polymorphism and ulcerative colitis: a mitochondria-wide genetic association study. *BMC Gastroenterol.* 16, 118. 10.1186/s12876-016-0509-1 27716073 PMC5048482

[DMM050895C10] de Goede, K. E., Harber, K. J. and Van DEN Bossche, J. (2019). Let's enter the wonderful world of immunometabolites. *Trends Endocrinol. Metab.* 30, 329-331. 10.1016/j.tem.2019.03.004 31060882

[DMM050895C11] Diskin, C., Ryan, T. A. J. and O'neill, L. A. J. (2021). Modification of proteins by metabolites in immunity. *Immunity* 54, 19-31. 10.1016/j.immuni.2020.09.014 33220233

[DMM050895C12] Fremder, M., Kim, S. W., Khamaysi, A., Shimshilashvili, L., Eini-Rider, H., Park, I. S., Hadad, U., Cheon, J. H. and Ohana, E. (2021). A transepithelial pathway delivers succinate to macrophages, thus perpetuating their pro-inflammatory metabolic state. *Cell Rep.* 36, 109521. 10.1016/j.celrep.2021.109521 34380041

[DMM050895C52] Friedrich, M., Pohin, M. and Powrie, F. (2019). Cytokine networks in the pathophysiology of inflammatory bowel disease. *Immunity* 50, 992-1006. 10.1016/j.immuni.2019.03.017 30995511

[DMM050895C53] Gwyer-Findlay, E., Sutton, G. and Ho, G. T. (2021). The MARVEL trial: a phase 2b randomised placebo-controlled trial of oral MitoQ in moderate ulcerative colitis. Immunother. Adv. 1, 1-4. 10.1093/immadv/ltaa002 PMC9585668 36284899

[DMM050895C13] Haberman, Y., Karns, R., Dexheimer, P. J., Schirmer, M., Somekh, J., Jurickova, I., Braun, T., Novak, E., Bauman, L., Collins, M. H. et al. (2019). Ulcerative colitis mucosal transcriptomes reveal mitochondriopathy and personalized mechanisms underlying disease severity and treatment response. *Nat. Commun.* 10, 38. 10.1038/s41467-018-07841-3 30604764 PMC6318335

[DMM050895C14] Hegarty, L. M., Jones, G. R. and Bain, C. C. (2023). Macrophages in intestinal homeostasis and inflammatory bowel disease. *Nat. Rev. Gastroenterol Hepatol.* 20, 538-553. 10.1038/s41575-023-00769-0 37069320

[DMM050895C15] Ho, G. T., Aird, R. E., Liu, B., Boyapati, R. K., Kennedy, N. A., Dorward, D. A., Noble, C. L., Shimizu, T., Carter, R. N., Chew, E. T. S. et al. (2018). MDR1 deficiency impairs mitochondrial homeostasis and promotes intestinal inflammation. *Mucosal. Immunol.* 11, 120-130. 10.1038/mi.2017.31 28401939 PMC5510721

[DMM050895C16] Ho, G. T. and Theiss, A. L. (2022). Mitochondria and inflammatory bowel diseases: toward a stratified therapeutic intervention. *Annu. Rev. Physiol.* 84, 435-459. 10.1146/annurev-physiol-060821-083306 34614372 PMC8992742

[DMM050895C50] Hsu, Y., Nayar, S., Gettler, K., Talware, S., Giri, M., Alter, I., Argmann, C., Sabic, K., Thin, T.H., Ko, H.-B.M. et al. (2023) NOX1 is essential for TNFa-induced intestinal epithelial ROS secretion and inhibits M cell signatures. *Gut* 72, 654-662. 10.1136/gutjnl-2021-326305 36191961 PMC9998338

[DMM050895C17] Huang, C., Wang, J., Liu, H., Huang, R., Yan, X., Song, M., Tan, G. and Zhi, F. (2022). Ketone body beta-hydroxybutyrate ameliorates colitis by promoting M2 macrophage polarization through the STAT6-dependent signaling pathway. *BMC Med.* 20, 148. 10.1186/s12916-022-02352-x 35422042 PMC9011974

[DMM050895C18] Jackson, D. N., Panopoulos, M., Neumann, W. L., Turner, K., Cantarel, B. L., Thompson-Snipes, L., Dassopoulos, T., Feagins, L. A., Souza, R. F., Mills, J. C. et al. (2020). Mitochondrial dysfunction during loss of prohibitin 1 triggers Paneth cell defects and ileitis. *Gut* 69, 1928-1938. 10.1136/gutjnl-2019-319523 32111635 PMC7483170

[DMM050895C19] Johnson, M. O., Wolf, M. M., Madden, M. Z., Andrejeva, G., Sugiura, A., Contreras, D. C., Maseda, D., Liberti, M. V., Paz, K., Kishton, R. J. et al. (2018). Distinct regulation of Th17 and Th1 cell differentiation by glutaminase-dependent metabolism. *Cell* 175, 1780-1795.e19. 10.1016/j.cell.2018.10.001 30392958 PMC6361668

[DMM050895C20] Kolho, K. L., Pessia, A., Jaakkola, T., De Vos, W. M. and Velagapudi, V. (2017). Faecal and Serum metabolomics in paediatric inflammatory bowel disease. *J. Crohns Colitis* 11, 321-334. 10.1093/ecco-jcc/jjx002.602 27609529

[DMM050895C21] Lampropoulou, V., Sergushichev, A., Bambouskova, M., Nair, S., Vincent, E. E., Loginicheva, E., Cervantes-Barragan, L., Ma, X., Huang, S. C., Griss, T. et al. (2016). Itaconate links inhibition of succinate dehydrogenase with macrophage metabolic remodeling and regulation of inflammation. *Cell Metab.* 24, 158-166. 10.1016/j.cmet.2016.06.004 27374498 PMC5108454

[DMM050895C22] Leber, A., Hontecillas, R., Tubau-Juni, N., Zoccoli-Rodriguez, V., Hulver, M., Mcmillan, R., Eden, K., Allen, I. C. and Bassaganya-Riera, J. (2017). NLRX1 regulates effector and metabolic functions of CD4^+^ T cells. *J. Immunol.* 198, 2260-2268. 10.4049/jimmunol.1601547 28159898 PMC5340590

[DMM050895C23] Leber, A., Hontecillas, R., Tubau-Juni, N., Zoccoli-Rodriguez, V., Abedi, V. and Bassaganya-Riera, J. (2018). NLRX1 modulates immunometabolic mechanisms controlling the host-gut microbiota interactions during inflammatory bowel disease. *Front. Immunol.* 9, 363. 10.3389/fimmu.2018.00363 29535731 PMC5834749

[DMM050895C24] Leber, A., Hontecillas, R., Zoccoli-Rodriguez, V., Bienert, C., Chauhan, J. and Bassaganya-Riera, J. (2019). Activation of NLRX1 by NX-13 alleviates inflammatory bowel disease through immunometabolic mechanisms in CD4^+^ T cells. *J. Immunol.* 203, 3407-3415. 10.4049/jimmunol.1900364 31694910 PMC6904519

[DMM050895C25] Lood, C., Blanco, L. P., Purmalek, M. M., Carmona-Rivera, C., De Ravin, S. S., Smith, C. K., Malech, H. L., Ledbetter, J. A., Elkon, K. B. and Kaplan, M. J. (2016). Neutrophil extracellular traps enriched in oxidized mitochondrial DNA are interferogenic and contribute to lupus-like disease. *Nat. Med.* 22, 146-153. 10.1038/nm.4027 26779811 PMC4742415

[DMM050895C26] Macias-Ceja, D. C., Ortiz-Masia, D., Salvador, P., Gisbert-Ferrandiz, L., Hernandez, C., Hausmann, M., Rogler, G., Esplugues, J. V., Hinojosa, J., Alos, R. et al. (2019). Succinate receptor mediates intestinal inflammation and fibrosis. *Mucosal Immunol.* 12, 178-187. 10.1038/s41385-018-0087-3 30279517

[DMM050895C27] Mancini, N. L., Goudie, L., Xu, W., Sabouny, R., Rajeev, S., Wang, A., Esquerre, N., Al Rajabi, A., Jayme, T. S., Van Tilburg Bernandes, E. et al. (2020). Perturbed mitochondrial dynamics is a novel feature of colitis that can be targeted to lessen disease. *Cell Mol. Gastroenterol. Hepatol.* 10, 287-307. 10.1016/j.jcmgh.2020.04.004 32298841 PMC7327843

[DMM050895C49] Mann, E. R., Lam, Y.K. and Uhlig, H. H. (2024) Short-chain fatty acids: linking diet the microbiomed immunity. *Nat. Rev. Immunol.* 24, 577-595. 10.1038/s41577-024-01014-8 38565643

[DMM050895C28] Michelucci, A., Cordes, T., Ghelfi, J., Pailot, A., Reiling, N., Goldmann, O., Binz, T., Wegner, A., Tallam, A., Rausell, A. et al. (2013). Immune-responsive gene 1 protein links metabolism to immunity by catalyzing itaconic acid production. *Proc. Natl. Acad. Sci. USA* 110, 7820-7825. 10.1073/pnas.1218599110 23610393 PMC3651434

[DMM050895C29] Monzel, A. S., Enriquez, J. A. and Picard, M. (2023). Multifaceted mitochondria: moving mitochondrial science beyond function and dysfunction. *Nat. Metab.* 5, 546-562. 10.1038/s42255-023-00783-1 37100996 PMC10427836

[DMM050895C30] Mottawea, W., Chiang, C. K., Muhlbauer, M., Starr, A. E., Butcher, J., Abujamel, T., Deeke, S. A., Brandel, A., Zhou, H., Shokralla, S. et al. (2016). Altered intestinal microbiota-host mitochondria crosstalk in new onset Crohn's disease. *Nat. Commun.* 7, 13419. 10.1038/ncomms13419 27876802 PMC5122959

[DMM050895C31] Murphy, M. P. and O'neill, L. A. J. (2018). Krebs cycle reimagined: the emerging roles of succinate and itaconate as signal transducers. *Cell* 174, 780-784. 10.1016/j.cell.2018.07.030 30096309

[DMM050895C32] Murphy, M. P. and O'neill, L. A. J. (2024). A break in mitochondrial endosymbiosis as a basis for inflammatory diseases. *Nature* 626, 271-279. 10.1038/s41586-023-06866-z 38326590

[DMM050895C33] Naujoks, J., Tabeling, C., Dill, B. D., Hoffmann, C., Brown, A. S., Kunze, M., Kempa, S., Peter, A., Mollenkopf, H. J., Dorhoi, A. et al. (2016). IFNs Modify the proteome of legionella-containing vacuoles and restrict infection Via IRG1-derived itaconic acid. *PLoS Pathog.* 12, e1005408. 10.1371/journal.ppat.1005408 26829557 PMC4734697

[DMM050895C34] Nunnari, J. and Suomalainen, A. (2012). Mitochondria: in sickness and in health. *Cell* 148, 1145-1159. 10.1016/j.cell.2012.02.035 22424226 PMC5381524

[DMM050895C35] Parikh, K., Antanaviciute, A., Fawkner-Corbett, D., Jagielowicz, M., Aulicino, A., Lagerholm, C., Davis, S., Kinchen, J., Chen, H. H., Alham, N. K. et al. (2019). Colonic epithelial cell diversity in health and inflammatory bowel disease. *Nature* 567, 49-55. 10.1038/s41586-019-0992-y 30814735

[DMM050895C36] Reina-Campos, M., Scharping, N. E. and Goldrath, A. W. (2021). CD8(+) T cell metabolism in infection and cancer. *Nat. Rev. Immunol.* 21, 718-738. 10.1038/s41577-021-00537-8 33981085 PMC8806153

[DMM050895C54] Rodriguez-Coleman, M. J., Schewe, M., Meerlo, M., Stigter, E., Gerrits, J., Pras-Raves, M., Sacchetti, A., Hornsveld, M., Oost, K. C., Snippert, H. J. et al. (2017). Interplay between metabolic identities in the intestinal crypt supports stem cell function. *Nature* 543, 424-427. 10.1038/nature21673 28273069

[DMM050895C37] Rutherford, D. and Ho, G. T. (2023). Therapeutic potential of human intestinal organoids in tissue repair approaches in inflammatory bowel diseases. *Inflamm. Bowel Dis.* 29, 1488-1498. 10.1093/ibd/izad044 37094358 PMC10472753

[DMM050895C38] Saxton, R. A. and Sabatini, D. M. (2017). mTOR signaling in growth, metabolism, and disease. *Cell* 169, 361-371. 10.1016/j.cell.2017.03.035 28388417

[DMM050895C39] Schett, G., Mcinnes, I. B. and Neurath, M. F. (2021). Reframing immune-mediated inflammatory diseases through signature cytokine hubs. *N. Engl. J. Med.* 385, 628-639. 10.1056/NEJMra1909094 34379924

[DMM050895C40] Sena, L. A., Li, S., Jairaman, A., Prakriya, M., Ezponda, T., Hildeman, D. A., Wang, C. R., Schumacker, P. T., Licht, J. D., Perlman, H. et al. (2013). Mitochondria are required for antigen-specific T cell activation through reactive oxygen species signaling. *Immunity* 38, 225-236. 10.1016/j.immuni.2012.10.020 23415911 PMC3582741

[DMM050895C41] Smillie, C. S., Biton, M., Ordovas-Montanes, J., Sullivan, K. M., Burgin, G., Graham, D. B., Herbst, R. H., Rogel, N., Slyper, M., Waldman, J. et al. (2019). Intra- and inter-cellular rewiring of the human colon during ulcerative colitis. *Cell* 178, 714-730.e22. 10.1016/j.cell.2019.06.029 31348891 PMC6662628

[DMM050895C42] Smith, A. L., Whitehall, J. C., Bradshaw, C., Gay, D., Robertson, F., Blain, A. P., Hudson, G., Pyle, A., Houghton, D., Hunt, M. et al. (2020). Age-associated mitochondrial DNA mutations cause metabolic remodelling that contributes to accelerated intestinal tumorigenesis. *Nat. Cancer* 1, 976-989. 10.1038/s43018-020-00112-5 33073241 PMC7116185

[DMM050895C43] Tannahill, G. M., Curtis, A. M., Adamik, J., Palsson-Mcdermott, E. M., Mcgettrick, A. F., Goel, G., Frezza, C., Bernard, N. J., Kelly, B., Foley, N. H. et al. (2013). Succinate is an inflammatory signal that induces IL-1beta through HIF-1alpha. *Nature* 496, 238-242. 10.1038/nature11986 23535595 PMC4031686

[DMM050895C44] Voss, K., Hong, H. S., Bader, J. E., Sugiura, A., Lyssiotis, C. A. and Rathmell, J. C. (2021). A guide to interrogating immunometabolism. *Nat. Rev. Immunol.* 21, 637-652. 10.1038/s41577-021-00529-8 33859379 PMC8478710

[DMM050895C45] Wang, R., Li, Z., Liu, S. and Zhang, D. (2023). Global, regional and national burden of inflammatory bowel disease in 204 countries and territories from 1990 to 2019: a systematic analysis based on the Global Burden of Disease Study 2019. *BMJ Open* 13, e065186. 10.1136/bmjopen-2022-065186 PMC10069527 36977543

[DMM050895C46] Zhang, Q., Raoof, M., Chen, Y., Sumi, Y., Sursal, T., Junger, W., Brohi, K., Itagaki, K. and Hauser, C. J. (2010). Circulating mitochondrial DAMPs cause inflammatory responses to injury. *Nature* 464, 104-107. 10.1038/nature08780 20203610 PMC2843437

